# The Effect of Psychological Hotwash on Resilience of Emergency Medical Services Personnel

**DOI:** 10.1155/2021/4392996

**Published:** 2021-08-20

**Authors:** Abbasali Ebrahimian, Seyed-Mahdi Esmaeili, Arash Seidabadi, Ali Fakhr-Movahedi

**Affiliations:** ^1^Clinical Research Development Center, Shahid Beheshti Hospital, Qom University of Medical Sciences, Qom, Iran; ^2^Nursing Care Research Center, Semnan University of Medical Sciences, Semnan, Iran; ^3^Student Research Committee, Nursing School, Semnan University of Medical Sciences, Semnan, Iran; ^4^Emergency Medicine Department, Shahroud University of Medical Sciences, Shahroud, Iran

## Abstract

**Introduction:**

Emergency medical services (EMS) personnel are exposed to stress. Job stress in EMS personnel can reduce their resilience and have adverse effects on their clinical performance and mental health, thus reducing the quality of their work. The present research was performed to determine the effect of psychological hotwash on resilience of emergency medical services personnel.

**Methods:**

This study was a quasiexperimental. Sixty-four EMS personnel were randomly divided into two groups of hotwash and control. The psychological hotwash program was performed in the intervention group for a month based on the protocol; however, the control group continued their usual work and received no intervention. A day and six weeks after the psychological hotwash in the intervention group, the resilience of the EMS personnel was remeasured in both groups.

**Results:**

Before the intervention, the participants' mean resilience score was 138.37 ± 7.04 in the intervention group and 137.34 ± 8.48 in the control group. There was a statistically significant difference between the mean scores of resilience in the intervention and control groups a day after the intervention (*P*=0.003). There was no statistically significant difference between the mean scores of resilience in the intervention and control groups 6 weeks after the intervention (*P*=0.102).

**Conclusion:**

The EMS personnel's attendance at psychological hotwash sessions could increase their resilience. Nevertheless, the sessions should not be interrupted because the 6-week interruption of the sessions caused the nonsignificant scores of resilience in the hotwash and control groups. Hence, it is recommended to continue the investigation of the effects of hotwash on resilience, stress reduction, and job burnout reduction in EMS personnel by other researchers in different settings.

## 1. Introduction

Emergency medical services (EMS) is a society-based system that responds to the medical needs of casualties or patients with acute and emergency illnesses outside of healthcare centers and until transferring to a medical center [1, 2]. EMS personnel are at high risk for mental disorders, stress, job burnout, depression, and posttraumatic stress disorder (PTSD) due to exposure to traumatic events and job stress [3–5]. In a systematic review, Petrie et al. (2018) indicated that the prevalence of PTSD, depression, and anxiety was 11%, 15%, and 15% in EMS personnel, respectively [6]. The prevalence of PTSD was reported as 50.21% in the Iranian EMS personnel [7], which is much higher than the world average.

Studies indicate that EMS personnel are exposed to stress due to the embodiment of their own death, complexity of patients' clinical conditions, interruption of EMS provision, health hazards, interpersonal problems, interprofessional interactions, legal conflicts, dependence on patients, and exposure to unsafe situations [8, 9]. Job stress in EMS personnel can reduce their resilience and have adverse effects on their clinical performance and mental health, thus reducing the quality of their work [10, 11]. Hence, it is necessary to reduce stress and increase resilience in EMS personnel.

Resilience has become more important in the last two decades. It enables people to control life stressors. It is also a successful adaptation to adverse environmental conditions [12]. Studies indicate that EMS personnel resilience is not desirable, and it can be affected by factors, such as secondary traumatic stress, resilience, posttraumatic growth, and changes in outlook, grit, and stress [13–15].

Analgesics, sedatives, acupuncture and acupressure, yoga, massage therapy, spirituality enhancement, and aromatherapy are usually used to reduce stress and increase resilience against some mental disorders [16–18]. However, some of these methods are associated with side effects, and others may be unwelcome by EMS personnel. The need assessments indicate that EMS personnel require easier access to mental health and improved interemployee relationships [4]. Studies also indicate that EMS personnel use talking with colleagues and inner dialogue strategies to deal with job burnout and increase resilience in stressful situations [19, 20]. It implies that EMS personnel tend to discuss their problems with other people to resolve their internal conflicts. Therefore, it seems that the use of debriefing known as psychological hotwash in the field of accidents and disasters can reduce stress and increase resilience in EMS personnel.

Psychological debriefing is a method of preventing the onset of PTSD symptoms in various injuries using approaches, such as normalizing responses, modulating emotions, and the way of dealing with symptoms of the disorder in a group session [21]. The method was first used during World War II. It was then developed by Jeffery Mithell in 1983 to manage stress in critical events and termed Critical Incident Stress Debriefing (CISD) [22]. CISD has been used in several studies to reduce PTSD, increase the quality of life, and improve clinical performance [23, 24]. However, the extent of its impact on the resilience of EMS personnel is not clear. Therefore, the present research was performed to determine the effect of psychological hotwash on resilience of emergency medical services personnel.

## 2. Methods

### 2.1. Study Design, Setting, and Participants

The research was a quasiexperimental study on EMS personnel of Shahroud in Semnan province, Iran, as the statistical population. The urban population was 218628 people during sampling, and their emergency measures were carried out by 20 EMS stations. There were 7 to 9 technicians working in each EMS station. To select the participants in the study, we first wrote the names of these 20 stations on separate papers and placed them in a small cloth bag. A 7-year-old child was then asked to take out 12 pieces of paper separately from the bag. The station's name on the first paper was placed in the intervention group and the station's name on the second paper was put in the control group. Using this method, 6 stations were considered in the intervention group and 6 in the control group. Moreover, EMS technicians working in the EMS stations with the inclusion criteria were included in the study as a research sample.

Inclusion criteria are as follows:

1. Take care of at least one emergency patient for 24 hours before attending the hotwash session
2. One-year experience in EMS
3. Do not have known mental and physical disorders

Exclusion criteria are as follows:

1. Unwillingness to continue participating in this research
2. Absence from attending 8 sessions of psychological hotwashing

### 2.2. Sample Size Estimation

To estimate the sample size, the first two groups of 5 individuals were selected in a pilot study, for which the study protocol was implemented. Then, the mean ± standard deviation of the difference in resilience scores in the pretest stage was calculated and compared with the scores of 4 weeks and 6 weeks later. The obtained values were inserted into G-Power 3.1.9 considering a 5% alpha, 80% power, and an effect size of 0.77. Using this method, the necessary sample size was 28 per group. As it was necessary to include all personnel of a station in the study, we included more EMS personnel in the study.

### 2.3. Measures

The research tools in this study included demographic information questionnaires and an EMSRS questionnaire. Demographic information questionnaire included age, BMI, marital status, number of children, work experience, number of shifts per month, and number of night shifts per month. The EMSRS questionnaire had 31 questions to assess resilience in EMS personnel in Iran. The questionnaire assessed six factors of job motivation, communication challenges, social support, calmness at the incident scene, self-management or self-care, and consequences of stress. The questionnaire was designed as a 5-point Likert-type scale (1 = *never*, 2 = *rarely*, 3 = *sometimes*, 4 = *often*, and 5 = *always*) and with a score range of 31 to 155. The higher scores indicated more resilience, and lower scores revealed less resilience. The validity and reliability of the questionnaire were confirmed in the study of Ebadi et al. (Cronbach's alpha of 0.91 and intrapolar correlation coefficient of 0.85) [25].

### 2.4. Data Collection

Data collection was performed in the workplace of EMS personnel. To this end, qualified personnel in both groups individually responded to demographic information and EMSRS questionnaires without consulting with others. Thereafter, the psychological hotwash program was performed in the intervention group for a month based on the protocol; however, the control group continued their usual work and received no intervention. The psychological hotwash was done in the room of the director of Shahrud Medical Emergency and Accident Center at 8 AM every day and after the end of the night shift with the help of a psychologist. There were 7 to 10 participants in each session. Each of the eligible EMS personnel participated in at least 8 sessions of psychological hotwash. The first session lasted about 2 hours and between 70 and 90 minutes, later. A day and six weeks after the end of the one-month program of psychological hotwash in the intervention group, we remeasured the resilience of the EMS personnel in both groups. The 6-week reexamination was considered for the participants' resilience based on the results of Salyers et al. [26].

#### 2.4.1. Study Protocol

The study was performed within 7 stages:

1. Preliminary stage: (a) acknowledgment of the presence of EMS personnel in session, (b) stating the objectives of the session, including training, quality improvement, and emotional processing, (c) explaining that the session was not a blame session, (d) explaining that the session lasted more than 60 minutes, and anyone could leave the session sooner for a personal reason, (e) creating a platform for all group members to speak and participate by asking them to introduce themselves; and (f) explaining that each participant can make suggestions to improve the quality of hotwash sessions.
2. Reality stage: at this stage, the participants were asked to talk about what happened in their previous shift. The EMS manager and a psychologist helped participants to recall the events by asking additional questions.
3. Thinking stage: at this stage, the participants talked about how to think about the problems that occurred in the previous shift and the meaning created by the problems in their minds.
4. Reaction stage: the participants' emotional responses to the events that occurred in the previous shift were examined.
5. Symptom stage: here, other reactions of the participants were examined (e.g. aggression, anger, change in appetite, headache, lethargy, confusion, lower accuracy, change in driving style, and sleep disturbance) to the events that occurred in the previous shift and their impact on their personal lives.
6. Training stage: at this phase, the EMS center manager and psychologist taught the participants regarding the coping strategies and the way of proper response to incidents.
7. Re-entry stage: at this stage, the process was reviewed quickly by the EMS manager or psychologist within 1 to 3 minutes, and the final result of the session was stated. The participants were asked to return to their normal lives [22].

### 2.5. Data Analysis

We entered data into the IBM SPSS version 16.0 software after collection and used descriptive statistics to indicate the frequency distribution, mean, and standard deviation of the data.

We used the independent *t*-test to compare age, BMI, number of children, work experience, number of shifts per month, and number of night shifts per month in the intervention and control groups. The chi-square test was used to compare marital status in the experimental and control groups.

The independent *t*-test was used to compare the resilience status between the intervention and control groups before and after the intervention and 6 weeks after the intervention. The significance level of all tests was 0.05.

### 2.6. Ethical Consideration

This study was approved by the Ethical Committee of Semnan University of Medical Sciences (Cod: IR.SEMUMS.REC.1398.240). Necessary coordination was made with the incident manager and the officials of the sampling sites. Then, the working method was explained to all EMS employees, and informed consent was obtained from them to participate in the research.

## 3. Results

**Figure 1:**
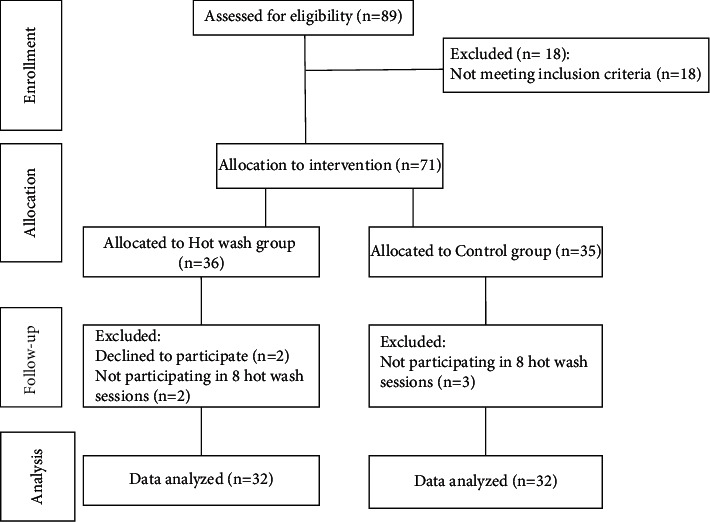
The flow of patients through the trial.

Among 89 EMS technicians, 71 met the inclusion criteria. However, 7 were excluded from the study for different reasons, such as not participating in 8 hotwash sessions (*n* = 5) and unwillingness to continue participating in the study (*n* = 2). Totally, 64 participants were randomly divided into two groups of intervention (*n* = 32) and control (*n* = 32), and their data were analyzed (Figure 1).

All participants in the study were male because all emergency medical technicians are male in Iran. The mean age of the participants was 35.00 ± 6.15. The minimum and maximum ages of the participants were 22 and 35 years, respectively. The participants' BMI was within the range of 18.90 to 33.95 and with an average of 25.36 ± 3.21. Among the participants, 82.80% were married and the rest were single. The EMS personnel's work experience was between 2 and 25 years with an average of 10.55 ± 5.29. The participants' average number of shifts per month was 17.58 ± 3.00, and the average number of night shifts was 8.61 ± 1.76. The average number of emergency missions was 10.68 ± 2.96 during the study. There was a statistically significant difference between age and BMI of the participants in the two groups (*P* > 0.05); however, the two groups were homogeneous in terms of marital status, work experience, number of shifts per month, number of night shifts per month, and number of emergency missions during the study (*P* > 0.05) (Table 1).

Before the intervention, the participants' mean resilience score was 138.37 ± 7.04 in the intervention group and 137.34 ± 8.48 in the control group. The statistical independent *t*-test indicated that there was no statistically significant difference between mean scores of resilience in the intervention and control groups before the intervention (*P*=0.498). A day after the end of the interventions, the participants' mean score of resilience was 140.71 ± 8.39 and 135.15 ± 6.05 in the intervention and the control groups, respectively. There was a statistically significant difference between the mean scores of resilience in the intervention and control groups a day after the intervention (*P*=0.003). The difference in resilience score changes was 5.22 ± 1.81 in the hotwash group and 2.18 ± 4.63 in the control group before and a day after the intervention. There was a statistically significant difference between the differences in the mean resilience scores of the intervention and control groups a day after the intervention (*P*=0.002) (Table 2).

Six weeks after the end of the interventions, the participants' mean score of resilience was 142.68 ± 7.58 and 139.68 ± 6.87 in the intervention and the control groups, respectively. There was no statistically significant difference between the mean scores of resilience in the intervention and control groups 6 weeks after the intervention (*P*=0.102). The difference in resilience score changes was 3.787 ± 4.13 in the hotwash group and 2.34 ± 4.17 in the control group before and six weeks after the intervention. There was no statistically significant difference between the differences in the mean resilience scores of the intervention and control groups six weeks after the intervention (*P*=0.171) (Table 2).

There was a significant difference between mean scores of job motivation, self-management, and social support subscales before and a day after the intervention (*P* ≤ 0.05). There was also a significant difference between the mean scores of the social support subscale before and six weeks after the intervention (*P* ≤ 0.05) (Table 3).

## 4. Discussion

In recent years, increasing resilience has attracted the focus of disaster managers [27]. EMS personnel experience a huge deal of stress while caring for patients, which can affect their clinical performance and decisions [14]. Increasing resilience can reduce stress, delay job burnout, and increase EMS personnel efficiency [13, 28]. Psychological hotwash is a way to help EMS personnel increase their resilience to job stress. The aim of this study was to determine the effect of psychological hotwash on resilience of emergency medical services personnel.

In this study, there was a statistically significant difference between the mean scores of resilience in the intervention and control groups a day after the end of the intervention. However, there was no significant difference between the mean scores of resilience in the intervention and control groups 6 weeks after interventions. The finding indicated that the degree of resilience increased in the EMS personnel attending hotwash sessions at least 8 times per month. However, if attending hotwash sessions was stopped for 6 weeks, the effect of hotwash on resilience would stop. Froutan et al. in a study found that a stress management program could reduce anxiety and increase EMS personnel's resilience [29]. Arce Edgar Carlos also found that the use of debriefing was effective in reducing the early respondents' stress [30]. Bohström et al. indicated that discussions with colleagues directly could reduce stress in EMS personnel [31]. In contrast, Woods and Ginger Lee argued that the EMS personnel's attendance at hotwash sessions did not help to treat PTSD symptoms and could even make it worse. He mentioned that the EMS personnel's attendance only in a debriefing session and using only a debriefing technique can worsen the symptoms of PTSD [32]. Devilly and Cotton also indicated that the use of debriefing was not appropriate to reduce workplace stress [33]. It seems that the noneffect of debriefing on stress in other studies was probably caused by the implementation of the debriefing protocol and its duration. Only a debriefing session was held in studies where debriefing did not affect stress. However, in this study, the debriefing sessions lasted for a month, and the EMS personnel attended at least 8 debriefing sessions per month. In addition to the presence of a psychologist, the manager of the Accident and Emergency Medical Center of the province also attended the session in our study. Therefore, it is suggested to continue the sessions and the presence of the EMS senior manager in the sessions for higher effectiveness of the debriefing protocol to reduce stress and increase resilience in EMS personnel. It is also recommended that a debriefing session be held after each heavy mission to reduce the stress of prehospital emergency personnel.

## 5. Conclusion

The EMS personnel's attendance at psychological hotwash sessions could increase their resilience. Nevertheless, the sessions should not be interrupted because the 6-week interruption of the sessions caused the nonsignificant scores of resilience in the hotwash and control groups. Hence, is recommended to continue the investigation of the effects of hotwash on resilience, stress reduction, and job burnout reduction in EMS personnel by other researchers in different settings.

## Figures and Tables

**Table 1:** Demographic and working status of psychological hotwash and control groups.

Variables	Frequency in groups			*P* value
	Hotwash	Control	Total	
Age (years), mean ± SD	36.63 ± 6.03	33.38 ± 5.92	35.00 ± 6.15	0.034^b^
BMI, mean ± SD	26.41 ± 2.99	24.33 ± 3.12	25.36 ± 3.21	0.003^b^

Marriage status, *n* (%)				
Married	29 (90.62)	24 (75)	53 (82.8)	0.098^c^
Single	3 (9.38)	8 (25)	11 (17.2)	
Work experience (years), mean ± SD	12.06 ± 5.81	9.03 ± 4.29	10.5 ± 5.29	0.086^b^
Number of shifts per month, mean ± SD	17.28 ± 3.00	17.88 ± 3.02	17.58 ± 3.00	0.385^b^
Number of night shifts per month, mean ± SD	8.22 ± 1.21	9.00 ± 2.12	8.61 ± 1.76	0.097^b^
Number of missions during the study, mean ± SD	80.56 ± 10.31	79.06 ± 10.13	80.20 ± 10.18	0.110^b^

Data are presented as *n* (%), or mean ± SD. SD: standard deviation. ^b^Independent samples *t*-test. ^c^Chi-square test.

**Table 2:** Comparison of the resiliency score before and after interventions in the psychological hotwash and control groups.

Times	Groups	Resiliency score		*t*	*df*	*P* value
		Mean	SD			
Before intervention	Hotwash	138.90	9.81	0.681	62	0.498^a^
	Control	137.34	8.47			

One day after the intervention	Hotwash	140.71	8.39	3.040	62	0.003^a^
	Control	135.15	6.05			

Six weeks after the intervention	Hotwash	142.68	7.55	1.660	62	0.102^a^
	Control	139.68	6.87			

The difference between resilience before the intervention and one day after the intervention	Hotwash	1.81	5.22	−3.23	62	0.002^a^
	Control	−2.18	4.63			

The difference between resilience before the intervention and six weeks after the intervention	Hotwash	3.78	4.13	−1.38	62	0.171^a^
	Control	2.34	4.17			

^**a**^Independent samples *t*-test.

**Table 3:** Comparison of the resiliency subscales mean scores before and after interventions in the psychological hotwash and control groups.

Resiliency subscales	Times	Groups	Resiliency score		*t*	*df*	*P* value
			Mean	SD			
Job motivation	Before intervention	Hotwash	58.62	5.02	0.354	62	0.725
		Control	58.21	4.11			
	One day after the intervention	Hotwash	60.50	2.99	2.103	62	0.040
		Control	58.68	3.84			
	Six weeks after the intervention	Hotwash	59.96	3.55	1.316	62	0.193
		Control	58.75	3.85			

Communication challenges	Before intervention	Hotwash	13.00	1.88	0.143	62	0.887
		Control	12.93	1.60			
	One day after the intervention	Hotwash	13.06	1.50	−0.774	62	0.442
		Control	13.34	1.40			
	Six weeks after the intervention	Hotwash	12.93	1.64	−1.135	62	0.261
		Control	13.37	1.43			

Social support	Before intervention	Hotwash	9.00	1.16	2.57	62	0.012
		Control	8.25	1.16			
	One day after the intervention	Hotwash	9.06	1.34	2.234	62	0.029
		Control	8.40	0.97			
	Six weeks after the intervention	Hotwash	9.18	1.02	3.110	62	0.003
		Control	8.40	0.97			

Remaining calm	Before intervention	Hotwash	22.56	1.96	0.246	62	0.806
		Control	22.43	2.09			
	One day after the intervention	Hotwash	23.53	1.52	0.637	62	0.526
		Control	23.31	1.20			
	Six weeks after the intervention	Hotwash	23.71	1.46	1.450	62	0.152
		Control	23.21	1.28			

Self-management	Before intervention	Hotwash	22.43	1.99	1.027	62	0.309
		Control	21.96	1.63			
	One day after the intervention	Hotwash	22.93	1.50	1.659	62	0.102
		Control	22.31	1.51			
	Six weeks after the intervention	Hotwash	22.96	1.46	1.920	62	0.059
		Control	22.25	1.52			

Consequences of stress	Before intervention	Hotwash	13.28	1.57	−0.614	62	0.541
		Control	13.53	1.68			
	One day after the intervention	Hotwash	13.96	1.03	0.809	62	0.422
		Control	13.68	1.67			
	Six weeks after the intervention	Hotwash	13.90	1.17	0.605	62	0.547
		Control	13.68	1.67			

## Data Availability

The data used to support the findings of this study are available from the corresponding author upon request.
